# Predicting Important Residues and Interaction Pathways in Proteins Using Gaussian Network Model: Binding and Stability of HLA Proteins

**DOI:** 10.1371/journal.pcbi.1000845

**Published:** 2010-07-08

**Authors:** Turkan Haliloglu, Ahmet Gul, Burak Erman

**Affiliations:** 1 Polymer Research Center, Bogazici University, Bebek, Istanbul, Turkey; 2 Division of Rheumatology, Department of Internal Medicine, Istanbul Faculty of Medicine, Istanbul University, Istanbul, Turkey; 3 Center for Computational Biology and Bioinformatics, Koc University, Istanbul, Turkey; National Cancer Institute, United States of America and Tel Aviv University, Israel

## Abstract

A statistical thermodynamics approach is proposed to determine structurally and functionally important residues in native proteins that are involved in energy exchange with a ligand and other residues along an interaction pathway. The structure-function relationships, ligand binding and allosteric activities of ten structures of HLA Class I proteins of the immune system are studied by the Gaussian Network Model. Five of these models are associated with inflammatory rheumatic disease and the remaining five are properly functioning. In the Gaussian Network Model, the protein structures are modeled as an elastic network where the inter-residue interactions are harmonic. Important residues and the interaction pathways in the proteins are identified by focusing on the largest eigenvalue of the residue interaction matrix. Predicted important residues match those known from previous experimental and clinical work. Graph perturbation is used to determine the response of the important residues along the interaction pathway. Differences in response patterns of the two sets of proteins are identified and their relations to disease are discussed.

## Author Summary

We propose a statistical thermodynamics model for determining structurally and functionally important residues in ligand-protein interactions. Our method identifies the path that the protein uses in transferring information from one point to the other. We show that a few energetically active residues are most efficient in energy exchange with the surroundings acting as ‘energy gates’. The remaining important residues that we identify are situated along the interaction path. These are the hub residues. Strong correlations exist between energy gates and hub residues along the interaction path, thus relating to allostery and cooperative binding. We studied the structure-function, ligand binding and allosteric activities of ten models of HLA Class I proteins of the immune system. Five of these models belong to the HLA-B*2705 allele and are strongly associated with a chronic inflammatory rheumatic disease. The remaining five from the HLA-B*2709 allele of the same protein are the corresponding properly functioning ones. We show that differences in the contact maps of the two types lead to significant and consistent changes in the fluctuation profile, making the HLA-B*2705 alleles respond too strongly to perturbation.

## Introduction

Transfer of information between two points in a protein is a fundamental problem relating to function [1], [2]. Fluctuations of residues in the native protein are the essential determinants of information transfer. The three dimensional native conformation or the topology of a protein determines the fluctuations of its residues. Relationships between topology and fluctuations offer important clues for the function of the protein. Structure-function relations can conveniently be understood by treating the protein as a graph of interacting residues [1]. Significant progress has been made in this direction over the past decade. Residue fluctuations, correlations, locations of conserved and important residues, stability of the native state, information transfer, energy fluctuations, and recently the protein-protein and protein-ligand binding have been studied by recourse to the graph-like state of the native topology [3]–[14]. The residue interaction graph contains important information in this respect that allows the determination of important interactions in a protein. The criticality of important interactions in a complex system is often the determining factor of stability of graphs [1], [15]. The lack of rearrangements in over determinate and highly constrained graphs result in decreased stability and robustness [15]. Our work here is centered on the determination of the subset of important interactions in proteins and the relationships between this set and function. We apply our analysis to a set of ten HLA class I proteins, HLA-B27, which are relevant examples of the relationship between critical interactions, robustness, and function.

The specific purpose of the present paper is to present a statistical thermodynamics model that gives a consistent explanation of structure-fluctuation-function relations in terms of the graph-like features of native proteins. We use the widely adopted Gaussian Network Model (GNM) based on a harmonic potential of residue-residue interactions, and propose a model for determining structurally and functionally important residues in relation to ligand-protein interactions as well as the path that the protein uses in transferring information form one point to the other. Our treatment is essentially an extension of the three recent papers [9]–[11] which we briefly summarize in the method section below in order to reduce cross-referencing. In the cited papers we showed, using statistical thermodynamics arguments, that the mode corresponding to the largest eigenvalue of the connectivity graph obtained from the contact map indicates the structurally and functionally important residues and that these residues are the ones for which energy and residue fluctuations are strongly correlated. We show that a few residues belong to the set of energetically active residues that are at the surface of the protein and are most efficient in energy exchange with the surroundings. We call these the ‘energy gates’. We also show that the residues that connect any such two surface residues along an interaction path are the ‘hub residues’ over which information is transmitted. From statistical mechanical arguments, a surface residue that is efficient in energy exchange with the surroundings is expected to be active in binding of a ligand, as the ligand-binding problem is an energy exchange problem. We also show that changes in the binding/interaction capacity of an energy gate or a hub residue changes the binding/interaction capacity of the other energy gate or hub residues. This has significant consequences relating to allostery and cooperative binding. The harmonic approximation that we adopt here is a coarse graining approach. However, many of the features obtained by this coarse graining are also indicated by more accurate treatments of protein behavior [16]–[19]. The GNM approach allows for a faster and easier visualization of structure-function relations.

We study the structure-function, ligand binding and allosteric activities of ten models of HLA-B27 Class I proteins of the immune system. Five of these models, which belong to the HLA-B*2705 allele of the HLA-B27 protein, are known to be strongly associated with a tendency to develop a chronic inflammatory rheumatic disease, known as ankylosing spondylitis, by causing yet unknown functional abnormalities. The remaining five are chosen from the HLA-B*2709 allele of the same protein. These are the corresponding properly functioning ones with almost no susceptibility for ankylosing spondylitis [20]–[31]. Each pair of the protein structures, one from the HLA-B*2705 and the other from the HLA-B*2709 allele, contains the same peptide in their antigen binding groove to present to immune cells, and therefore serves as an excellent benchmark to test the predictions of the GNM. The only difference between the B*2705 and B*2709 alleles is that residue 116 in the former is always an ASP, whereas it is HIS in the latter. This single residue difference between the two alleles causes structural differences in the two types, and therefore in their contact maps. We show that these differences in the contact map of the two types lead to significant and consistent changes in the fluctuation profile, making the members of the HLA-B*2705 allele respond too strongly to perturbation. Based on these changes, we propose a mechanism that is responsible in the functional differences of the two types.

## Methods

### The model and formulation of the problem

The system consists of the protein and its environment. The latter may contain ligands that are capable of binding to the protein. The protein and the environment form a closed system with fixed energy and amount of molecules. The protein exchanges energy with the environment.

Since the total energy of the protein and the surroundings is constant, we have
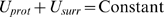
(1)

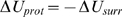
(2)where, *U_prot_* and *U_surr_* are the energies of the protein and the surroundings, respectively.

In the statistical thermodynamics treatment of proteins that we propose here, the thermodynamic variables for the protein are *S* = Entropy, *U* = energy, *V* = Volume of protein, ***R*** = Position of the residues. In the remainder of the paper, the thermodynamic variables are used for the protein only, without the subscript prot. The thermodynamic variables are averages. The instantaneous values of the energy, volume and residue positions are shown by 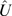, 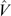, 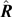, respectively. The fluctuations, 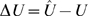, 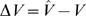, 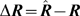 result from the deviations of the instantaneous extensive variables from their thermodynamic averages.

In the GNM model, the emphasis has been on the fluctuations 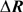, visualized as resulting from coupled harmonic motions of the residues from their mean positions [4]. The present treatment is based on the extension of the mechanistic description of the GNM to include the role of energy fluctuations, 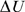, as well.

As in previous treatments, we adopt a coarse-grained model and represent each residue in terms of its alpha carbon. Thus, for a protein of n residues, 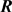 is defined as
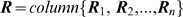
(3)Here, 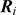 represents the Cartesian coordinates of the ith residue alpha carbon. 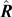 and 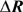 are similarly defined.

The probability 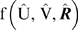 of the instantaneous values, 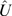, 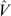, and 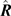, of the energy, volume and residue positions, is determined from the interrelation of the thermodynamic functions given in the Text S1. Therein, this probability function is used to derive the correlations between the fluctuations of residue positions and energy, as well as the cross correlations between the fluctuations of the energy and residue positions. The statistical thermodynamics interpretation of the GNM was given in full detail by Yogurtcu et al., [11], which was successfully applied to the prediction of binding sites in receptor-ligand complexes [10], of specific sites for binding [9]. In the present paper, we use the statistical thermodynamics approach to predict the important residues along an interaction pathway.

The starting point of the model is the equation relating fluctuations to thermodynamic averages. The derivation of this equation is given in the Text S1. We reproduce the resulting expression here to reduce cross-referencing
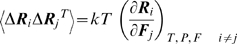
(4)Here, 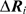 represents the position vector of the alpha carbon of the ith residue and the superscript T indicates the transpose. *k* is the Boltzmann constant, *T* is the temperature. 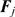 is the force on the jth alpha carbon. The subscripts of the parenthesis of the right hand side indicate that the temperature, pressure and the force on each residue except the ith is kept constant. Angular brackets indicate an average over all possible values of the argument. The right hand side is a thermodynamic quantity that expresses the change in the position of residues by the application of a force. The left hand side, on the other hand denotes an average of fluctuations. Thus, this equation relates fluctuations to average quantities. If fluctuations are associated with function, as is done in several previous studies [3], [6], [32], [33].

The right hand side of Eq. 4 requires the knowledge of a force-displacement relation. The simplest of such relations is that for the linear spring
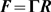
(5)where, **Γ** is the spring constant matrix. Multiplying both sides of Eq. 5 with the inverse of **Γ** and performing the differentiation shown in Eq. 4 leads to
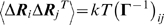
(6)In the GNM model, the **Γ** matrix is obtained by inserting a constant 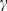 to the ij'th position if residues i and j are in contact and zero otherwise. Two residues are assumed to be in contact if they are separated by less than 7 Å. This value of the cutoff is approximately equal to the radius of the first coordination shell for residues in a protein. Each diagonal element of **Γ** is the negative sum of its row. In this way the protein is visualized as a graph, and the off-diagonal terms of **Γ** is the connectivity graph of the protein. Although the ij'th elements of the **Γ** matrix is set to zero when residues i and j are separated by more than 7Å, the ij'th element of the inverse matrix **Γ^−1^** is not zero. This implies correlations between residues that are not in contact. The correlation between the fluctuations of the ith and jth residues is determined by the full graph structure, since the ij'th element of the inverse of **Γ** contains contributions from all nodes of the graph, i.e., from all other residues, and not just from those in the close neighborhood of the ith and jth residues. As will be discussed in detail below, the essential features of the correlations may be understood largely by considering the largest eigenvalue of **Γ**.

Energy fluctuations of the protein are assumed to result from fluctuations of inter-residue interactions. The correlation 
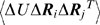
 of the energy fluctuations of the protein with fluctuations of residue positions is derived as (See Text S1)
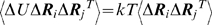
(7)The left hand side gives the correlations of energy fluctuations with the fluctuations of residues. The right hand side consists of correlations among residue fluctuations only. Writing the difference
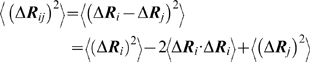
(8)and using the right hand side of Eq. 7 with the appropriate choice of the indices, we can write
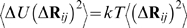
(9)For the case of harmonic fluctuations, i.e., GNM, this relation is derived in the Text S1.

If 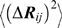 is assumed to represent the mean-square fluctuation in the ‘spring length’ connecting residues i and j, the left hand side of Eq. 4 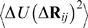 becomes proportional to the fraction of the incoming energy from the surroundings absorbed by the spring. The right hand side of Eq. 9 is positive. The terms in the angular brackets on the left hand side may be positive or negative depending on the sign of Δ*U*. But, for the average to be positive, there must be a constraint on the elements of the left hand side: Positive fluctuations of the energy, which indicates energy transfer into the protein from its surroundings, should couple to large values of 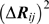 and negative fluctuations should couple to small values. Stated in another way, energy that is absorbed from the surroundings are stored in pairwise interactions between residues i and j according to Eq. 8.

In the application of the model to the HLA proteins, we define the variable 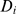 as the sum of the ith row of the correlation matrix
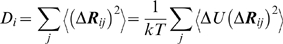
(10)A finite value of 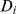 indicates that residue i belongs to the subset of energetically active residues that are either energy gates or lie along an interaction pathway. It also is a measure of the energy absorbed from the surroundings as may be seen from the second equality in Eq 10.

According to graph theory, important features of the graph, such as graph perturbation that relates to allostery for example, may be obtained by considering the largest eigenvalue and eigenvector of the graph [34]. The choice of the largest eigenvalue mode is specifically relevant, because (i) it corresponds to localized effects where only a few residues are excited [3] and (ii) the largest eigenvalue is the most sensitive to perturbation of the graph [34]. In the case that the residues identified by the highest mode are adjacent in space, then they interact and form a path that is active in long distance communication. Our calculations for a large number of ligand-protein systems show that the largest eigenvalue and the corresponding mode of the 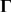 matrix is in general sufficient to point to the known functionally relevant residues. Within the present approximation, we adopt the maximum eigenvalue interpretation.

A residue at the surface with a large value of 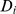 is an ‘energy gate’ through which the protein executes its energy interactions with the surroundings. If the residue with high 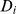 is not at the surface but inside the protein, then it is a ‘hub residue’ that has important function along the interaction pathway connecting to an energy gate. Although there is no proof, hubs are generally located between two energy gates in allosteric processes [35], [36]. Examples shown below are in support of this statement. For a residue i at the surface, 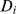 is a measure of whether residue i will interact with the ligand. For the hub residues, 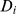 is a measure of the importance of that residue within the network of information exchange.

The quantity 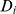 introduced above indicates the extent of correlation of the given residue i with the rest of the protein. Any change in the connectivity state of residue i will affect the behavior of the rest of the protein through the subset of energetically active residues. One way to apply this change would be to bind a ligand to i, and to the residues within the cutoff distance of i. This corresponds to perturbing the entries in the ith row and column of the **Γ** matrix. The relation of this to allosteric manipulation is obvious. In this section we discuss the changes 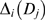 in the interaction energies of residues j when the parameters of residue i are modified. Binding to a point i on the protein may increase or decrease the residue interaction energy of other points.

The calculations and the analysis are carried out with respect to the heavy chain A, taking the structure from the complex structure of chains A, B (beta-2-microglobulin) and C (peptide bound on the antigen binding groove of chain A) in the alleles HLA-B*2705 and HLA-B*2709 alleles.

## Results

### Application to HLA proteins

Ten HLA-B27 protein structures are analyzed here. Five of the structures belong to the HLA-B*2705 allele and the remaining five belong to the HLA-B*2709 allele, where the residue 116 is ASP in the former and HIS in the latter. Each pair has the same peptide sequence bound. The PDB codes of the proteins and their alleles are presented in the first and third columns of Table 1.

**Table 1:** Energetically active residues of HLA B*2709 and B*2705 proteins suggested by GNM.

Protein (B*2709)	Residue	Protein (B*2705)	Residue
1OF2	101, 160, 6, 103, 164, 109, 5, 113, 27, 161,164, 168	1OGT	101, 6, 160, 103, 164, 27, 5, 113, 164, 168
1UXW	101, 160, 6, 103, 164, 109, 113, 5, 27, 161	1UXS	101, 103, 160, 6, 164, 27, 100, 5, 113, 124
1W0W	101, 6, 160, 164, 103, 165, 5, 109, 113, 27	1W0V	101, 6, 160, 103, 164, 165, 27, 5, 113, 168
1K5N	101, 160, 103, 6, 164, 168, 161, 5, 170, 113, 27	1JGE	101, 6, 160, 164, 103, 165, 5, 109, 113, 27
3BP7	101, 6, 160, 103, 164, 165, 27, 5, 113, 168	3BP4	101, 103, 160, 6, 164, 27, 5, 100, 113, 25

The residues are ordered according to their D_i_ values.

**Figure 1:**
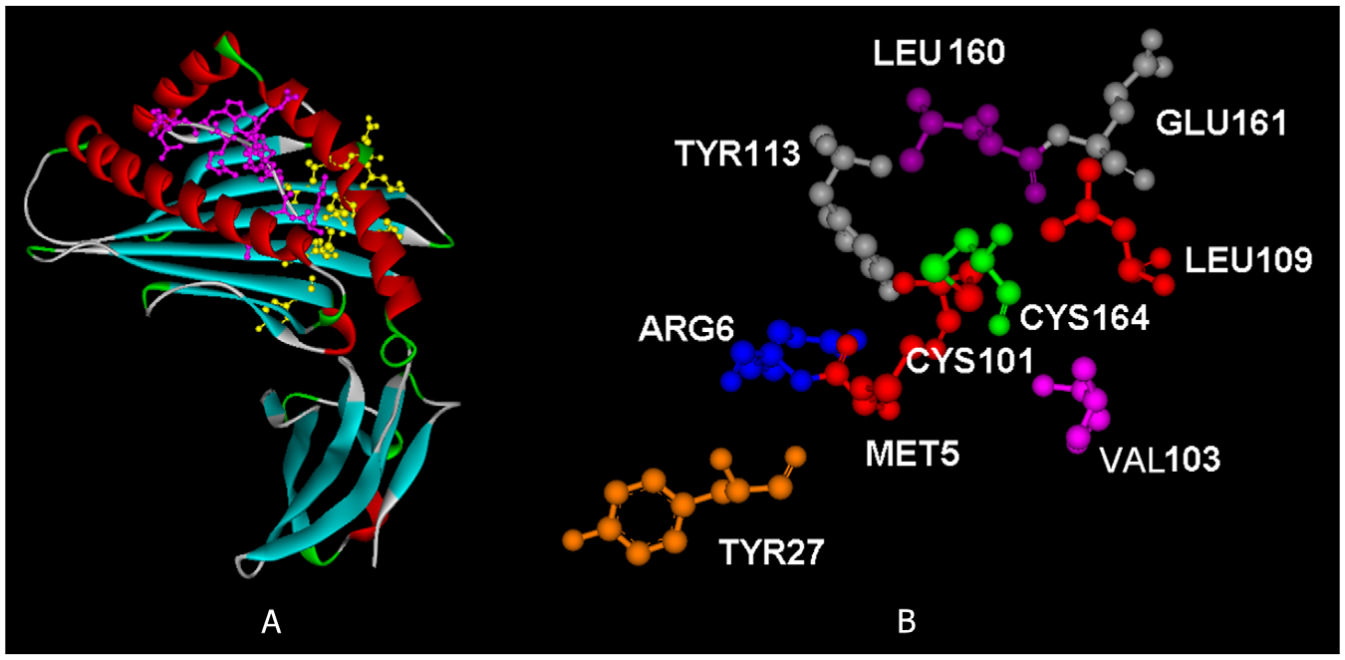
Solid ribbon diagram of the heavy chain (A) of HLA-B*2709 (1OF2.PDB). The ligand peptide is shown in indigo. The functionally important residues 6, 7, 27, 101 and 164 are indicated in yellow. Enlarged version showing only the important residues predicted (B).

In Figure 1A, the ribbon diagram of the heavy chain of 1OF2.PDB of the HLA-B*2709 allele is shown. The nine residue peptide is shown in indigo. It sits in the groove between the two helices shown in red. In the same figure, we show the positions of the energetically active and functionally important residues 6, 7, 27, 101 and 164 for 1OF2 in yellow, suggested by the GNM calculations. Figure 1B is an enlarged version of Figure 1A where each residue is shown with a different color and the rest of the protein is not shown. Figure 1B clearly shows the important residues that form an interaction pathway, with GLU161 at one end and TYR27 on the other end of this path. The prediction of these residues, and the detailed discussion of their role in the functioning and malfunctioning of the HLA-B*2709 and HLA-B*2705 will be given below.

In all of the calculations presented in this paper, we concentrate only on the largest eigenvalue which suffices for presenting a proof-of-principle discussion. A more detailed discussion may need to involve eigenvalues other than the largest, which might be plausible for additional functionally and structurally residues.

### Determination of the energy gates and hub residues of the HLA class I molecules

**Figure 2:**
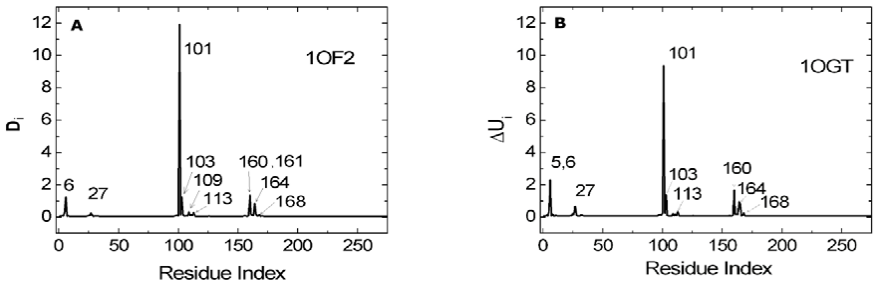
Important residues of (A) 1OF2 (B*2709) and (B) 1OGT (B*2705) predicted by GNM. The ordinate values are obtained from Eq. 10.

We use Eq. 7 (see Methods) to obtain the energetically active residues using the high frequency mode. In Figure 2, we show the 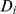 plots of the two proteins 1OF2 and 1OGT, where the subscript i indicates the residue index. The plots are obtained as follows: First, the **Γ** matrix is constructed with a cutoff distance of 7 Å and the correlations are calculated using Eq. 3. The components of the correlation matrix corresponding to the largest eigenvalues of the **Γ** matrix are determined by reconstructing the correlation matrix keeping the largest eigenvalue only, and the columns of the resulting matrix are added according to Eq. 10 in order to obtain the 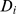 values presented in the figures.

Figure 2 displays that residue CYS101plays the most significant role in the interactions of the protein and has the strongest correlations with other residues such as ARG6, TYR27, LEU160 and CYS164 in both 1OF2 and 1OGT. This is also observed in the other HLA-B*2709 and HLA-B*2709 alleles. The residues with high 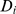 values that are observed in the ten proteins are shown in Table 1. The other feature observed that is in common for all of the ten proteins is that the 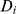 value for the 101st residue is always larger for the HLA-B*2709 alleles than for HLA-B*2705. Since these residues are calculated from the largest eigenvalue of the **Γ** matrix, we call them the important residues and show with the examples below that these play role in the stability and function of the protein.

From the definition given by Eq. 10, 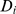 is the sum of the distance fluctuations of the intermolecular bonds which the ith residue makes with others. Equation 4 shows that 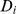 reflects the energetic interactions of residue i with other residues. In this sense, CYS101 acts as the central hub, which controls the system. There are two different types of terms on the right hand side of Eq. 8, the self terms 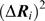, 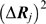 and the cross term 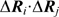. In order for 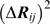 to be large, both 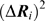 and 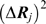 should be large, and 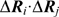 should be negative, i.e., residues i and j should make anti-correlated motions. Only in this case 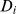 can be large and energy can be transferred from one to the other via the spring that connects them.

Figure 2 shows that there are essentially four groups of residues that are of significance: (i) residue 6, (ii) residue 27, (iii) residues 101–116, and (iv) residues 160–164. In the remaining sections, we elaborate on the characteristic features of these four groups of residues that are also observed in the other HLA-B*2709 and HLA-B*2709 alleles (Table 1).

### Perturbation of the residues on the interaction pathway

In this section, we study the differences of the response of the residues for a perturbation along the interaction pathway between the two families. These differences arise from the presence of the negatively charged ASP116 in HLA-B*2705 and positively charged HIS116 in HLA-B*2709 that induce energetic changes along the interaction pathway, resulting in functional differences. The differences will be outlined in the following sections. In this section, we present the results of our calculations based on Eq. 10.

**Figure 3:**
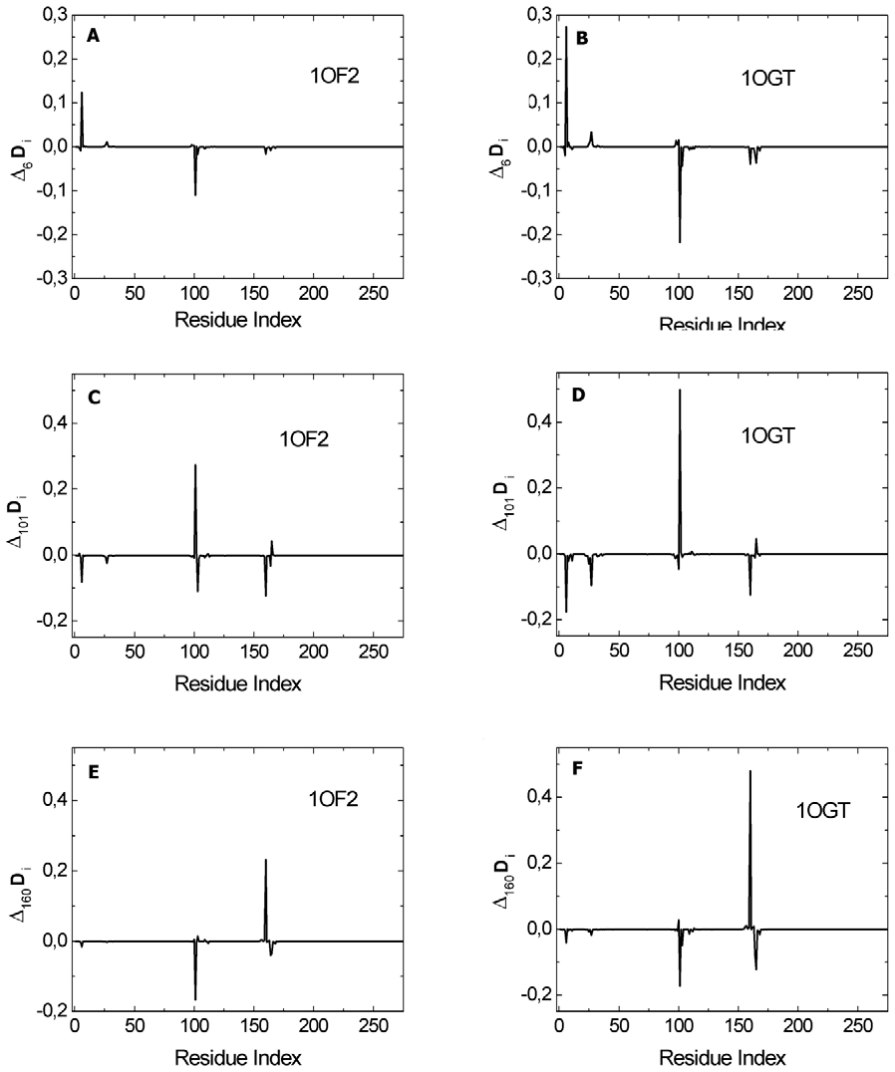
Comparison of the responses of 1OF2 (B*2709) and 1OGT (B*2705), where the perturbation is applied to residues (A) ARG6 of 1OF2, (B) ARG6 of 1OGT, (C) CYS101 of 1OF2, (D) CYS101 of 1OGT, (E) LEU160 of 1OF2, (F) LEU160 of 1OGT.

In the interest of observing the response of a protein to an external stimulus, we induce changes in the interaction strength of each important residue and observe the response of the remaining residues. This is done by increasing the interacting strength of contacts of the ith residue by 1%. This amounts to multiplying the off diagonal elements of the ith row and column of the **Γ** matrix by 1.01, and recording the difference 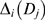 in the values of 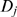 obtained after and before this perturbation for each residue j. In this notation Δ is the change in 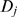, and the subscript i indicates that the perturbation is applied on the ith residue. A perturbation of 1% was chosen to ensure that the system was in the linear response region. Trial choice of values above 10% resulted in nonlinear response. In the linear response regime, changing the perturbation from 1% to 2%, for example, doubled the output. Figures 3A–F, given for the case of 1OF2-1OGT, show that the B*2705's respond to perturbations strongly compared to B*2709's. The other B*2705's and B*2709's, also show the same difference. This difference has its roots in the differences of residue-residue interaction energies. The residue ASP116 in B*2705's results in strong interactions with its surrounding residues, making the protein respond strongly to perturbations. An examination of Figure 3 shows that positive perturbation of ARG6 induces a decrease in the response of CYS101 which is stronger for all of the B*2705 alleles than for B*2709. It is worth noting that the residue that is directly involved in the binding of the ligand is TYR7. However, its perturbation does not result in any noticeable perturbation in the rest of the protein, suggesting that it does not directly lie on the interaction pathway. However, perturbation of its neighbors MET5 and ARG6 induces strong changes in the behavior of the protein. This is because in the native structure, the environment of TYR7 is less compact than that of ARG6. In this respect, ARG6 plays a special role in the pathway we identified. For example, mutating ARG6 into ALA6 in 1OGT (see the following section describing energy calculations) caused four times more energy increase then mutating TYR7 into ALA7. Based on this evidence we hypothesize that the direct interaction of the ligand with TYR7 induces a perturbation of ARG6 which affects the protein structure significantly.

**Figure 4:**
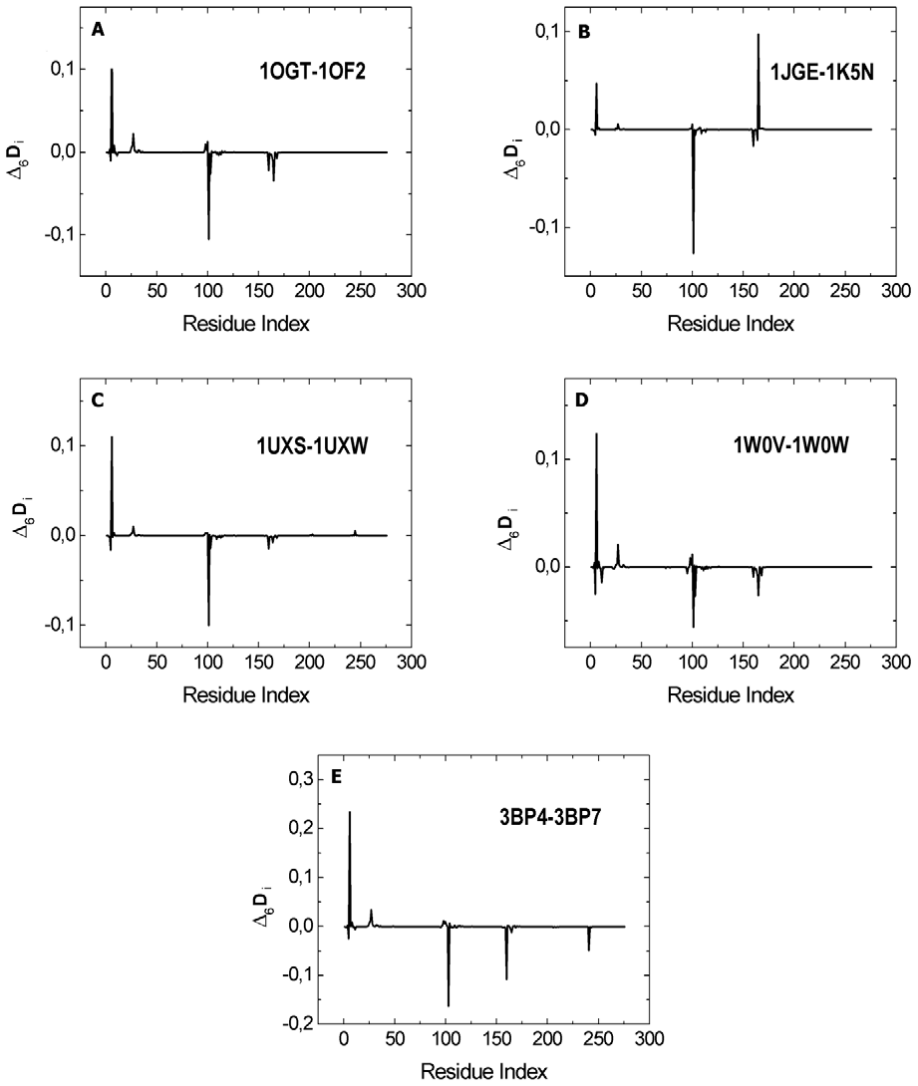
Differences in response against perturbation of ARG6 for the five pairs of HLA-B27 proteins; 1OGT-1OF2 (A), 1JGE-1K5N (B), 1UXS-1UXW (C), 1W0V-1W0W (D), 3BP4-3BP7 (E).

In order to see the differences in response of the two alleles, we subtracted the 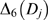 values of B*2709 from those of B*2705 for ARG6 for each allele pair, and presented the results in Figure 4. The figures show that although ARG6 is perturbed positively by the same amount for both alleles, i.e., the related elements of the **Γ** matrix are perturbed by 1% in both cases, the response of B*2705's is stronger at ARG6 and the CYS101 response to this perturbation is always negative, and stronger again in all B*2705's. Energy calculations show that the interactions of ARG6 with its environment is 9 kcal/mole stronger in B*2705 than in B*2709, which means that ARG6 is more rigidly embedded in its surroundings in B*2705. This is the result of the differences of residue 116 that affect the two alleles in different ways and the effects are seen on residues ARG6 and CYS101.

The only difference between the sequence of B*2705's and B*2709's is in the residue 116. This residue is located at the bottom of the B-pocket where the peptide binds. This single mutation is thought to cause differences in the stiffness of the structure around 116 that result in the differences observed and reported above for the two alleles [21]. In order to understand the energetic differences of the two alleles, we calculated the interaction energy of residue 116 with its surroundings in the two alleles.

### Results of comparative energy calculations

The knowledge of the interaction energies of specific residues in the system may be helpful for understanding and comparing the behavior of the two alleles under study. Here, we present approximate calculations of interaction energies obtained by static minimization of the energies, briefly described in Text S1. The energy minimization calculations are only for comparison of the B*1705 and B*2709, where we either compare two different systems, or compare two different situations on the same system. Thus, the relative values rather than the absolute values of the energies reported here are of interest here to have an estimate on the differences between the B*1705 and B*2709.

In our calculations, we first minimized the energy of the system. To calculate the interaction of a given residue with the rest of the protein at its minimum energy conformation, the residue is chosen in the matrix of the remaining residues that are kept in their native states. The interaction energy of the chosen residue is then minimized around the given conformation. In this calculation, only the residue of interest is left flexible and the conformations of the remaining protein residues are kept fixed at their native values. As the energies are sensitively dependent on the value of the dielectric constant chosen, different values of the dielectric constant are used to see the effect on the calculated energies. In the absence of explicit water, the value range of 1–4 is usually considered in the calculations of biological systems.

**Figure 5:**
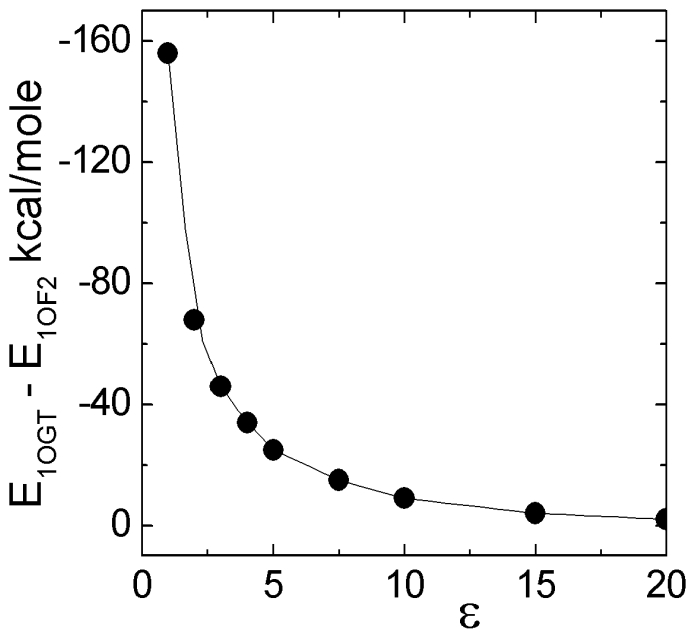
The interaction energy difference of residue 116 in 1OGT and 1OF2 calculated for different values of the dielectric constant ε.

We minimized the energy of 1OGT.PDB and 1OF2.PDB and calculated the energy of residue 116 in each structure as described in the preceding paragraph. We then removed the rest of the protein and minimized the energy of the isolated residue 116. The energy calculated in this way is the intra-residue energy for 116 and contains bond, bond angle, electrostatic, hydrogen bonded and nonbonded energies of the atoms that all belong to 116 only. The difference between the energy in the presence and absence of the surroundings gives an idea on how strongly the residue interacts with its neighbors in the protein. For the dielectric constant equal to unity, the energy of residue 116 is 156 kcal/mol lower in 1OGT (B*2705) because of the charge differences of the two residues, where HIS is positively charged with a pK of 6.5 while ASP is negatively charged with a pK of 3.1, which is the ‘random coil’ or ‘model compound’ small peptide pKa value. In 1OGT, the carbonyl group of the negatively charged ASP116 is within 2.9 Å of the positively charged amino end of LYS70, and within 5.5 Å of the positively charged HIS114, whereas in 1OF2 (B*2709), the positively charged HIS116 is 6 Å to the nearest negatively charged ASP122. In 1OGT, the two residues of the peptide binding site, ARG6 and ASN97 have lower energies in 1OGT compared to 1OF2. This means, these two residues are embedded strongly in their environments in 1OGT, and perturbing their states results in strong responses in the protein. The unrealistically high energy values reported here are upper bounds that are obtained by taking the dielectric constant as unity. We calculated the binding energies by varying the dielectric constant over a wide range. The results are presented in Figure 5. The difference between the two proteins vanishes when the dielectric constant is around 20. The realistic values of ɛ used for biological systems vary in the range 1–4. Even with a value of ε = 4, the energy difference is as high as 30 kcal/mole.

In Table 2, we present the differences in these energies for 1OGT (B*2705) and 1OF2 (B*2709) for residue 116 and for a few other residues with the values calculated for ε = 1. These values are the upper bounds. We see that GLU163 in 1OGT is bound to its neighborhood less strongly than the one in 1OF2 by an energy difference of 61.0 kcal/mole. This difference comes from the presence of the negatively charged LYS3 of the peptide in close vicinity of GLU163 in 1OF2. In the energy minimized structure, the oxygen of the carbonyl group of GLU163 is 2.3 Å from the hydrogen of the amino group of LYS3 of the peptide, whereas this distance is 5.6 Å in 1OGT. This interaction indicates the specificity of binding of the peptide to 1OF2, which is lacking in 1OGT.

**Table 2:** Energy differences between a few residues of the two alleles.

Residue	Energy difference 1OGT–1OF2 (kcal/mol) ε = 1
ARG6	−18
ASN97	−18
ASP/HIS116	−156
GLU163	61

In Table 3, differences in the interaction energy of the peptides of the two alleles and the energy of the residue GLU163 are presented. The approximations involved in these calculations are explained in the Text S1. In the second column, we compare the binding energy of the full peptide to the proteins. The values are the differences of the binding energies to alleles B*2705 and B*2709. Among the different residues, GLU163 exhibits a peculiar difference in that its energy is much lower in B*2709's. For this reason we calculated the difference in the energy of GLU163 for the two alleles and presented the results in the third column of Table 3. Although the overall binding energy of the peptide to B*2705 is more favorable in all cases, the energy of GLU163 is more favorably in B*2709.

**Table 3:** Differences in the interaction energies of the peptides to the two alleles.

	Binding energy of peptide (kcal/mol) ε = 1	Energy of GLU163 (kcal/mol) ε = 1
ΔE_1OGT_-ΔE_1OF2_	−54	61
ΔE_1UXS_-ΔE_1UXW_	−15	41
ΔE_1W0V_-ΔE_1W0W_	−19	31
ΔE_1JGE_-ΔE_1K5N_	−30	39
ΔE_3BP4_-ΔE_3BP7_	−9	30

It is worth noting that the computations by using the largest eigenvalue approach indicated LEU160 and CYS164 as the important residues and GLU163 does not appear as an important residue. A similar trend is observed for several other systems not reported here, where the maximum eigenvalue approach points to a close neighbor of an important residue as in the present study. The difference arises mostly from the presence of electrostatic interactions in the neighborhood of the important residue. Predictions by the maximum eigenvalue method do not directly consider the electrostatic interactions. However, their presence affects the topology that is reflected in the maximum eigenvalue method.

In the energy calculations described above, in order to see why ARG6 appeared in the interaction path, we mutated ARG6 into ALA6 in 1OGT, which resulted in fourfold increase in the energy of the system (See Text S1). Same calculations are performed by mutating TYR7 into ALA7.

These values, though approximate and relative, it consistently points to some important features of the system. Nevertheless, it is worth stating here that the appropriate and rigorous computational practice in computational biology is to perform an extensive molecular dynamics simulation of the protein and the ligand in aqueous medium and extract the required energies as thermodynamic averages, which may include the evaluation of the free energy as well. Our present energy minimization approach is only for exploratory purposes.

## Discussion

**Figure 6:**
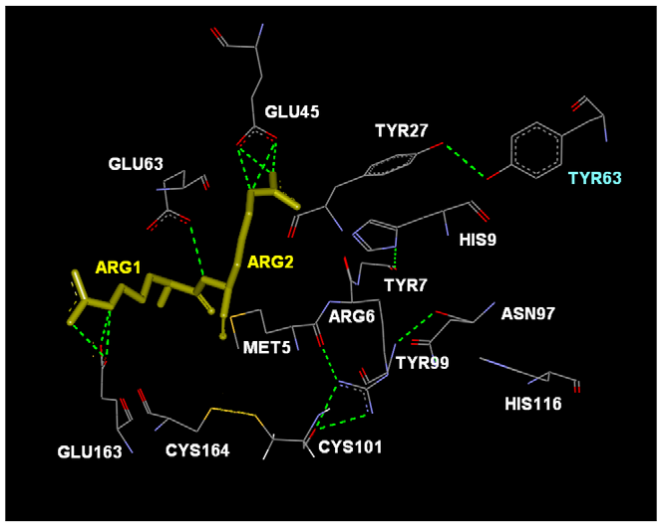
The important residues along the interaction pathway between TYR27 and CYS101 for 1OF2. The peptide is shown in yellow stick representation. The dotted green lines are the hydrogen bonds. TYR63 shown in indigo belongs to the light chain B.

**Figure 7:**
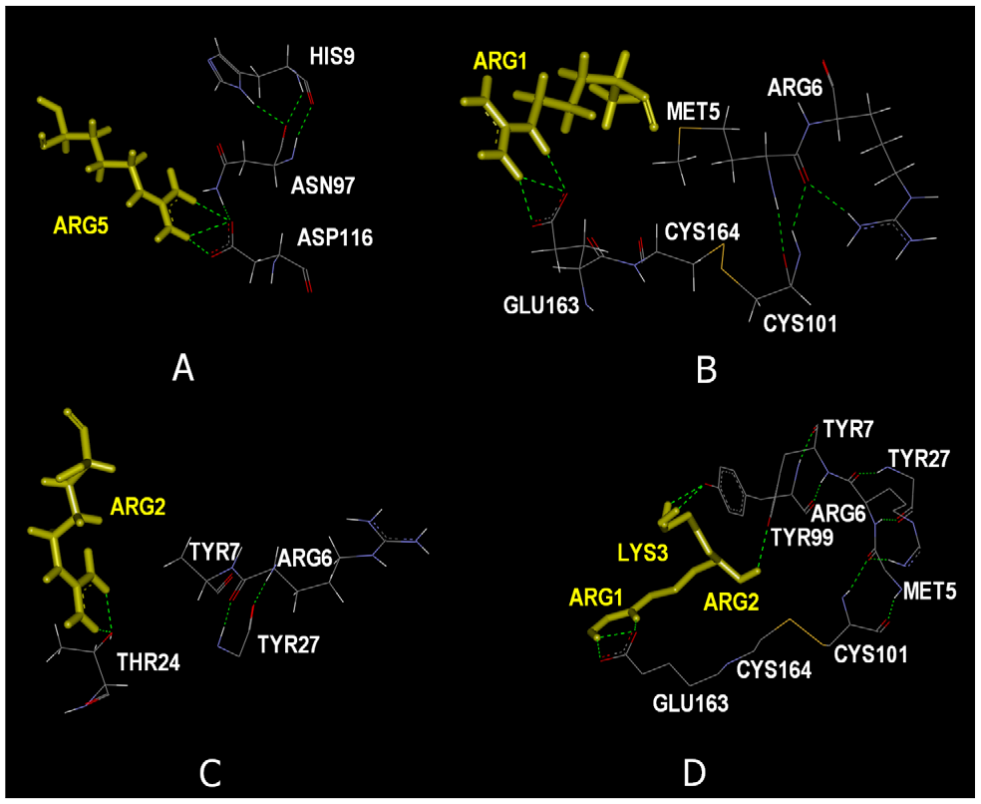
The important residues along the various interaction regions of 1OGT with respect to the peptide sites (shown in yellow stick representation): ARG5 (A), ARG1 (B), ARG2(C), ARG1-LYS3 (D). The dotted green lines are the hydrogen bonds.

We identified the interaction paths of the B*2705 and B*2709 alleles. This path contains the residues TYR27 at one end and CYS164 at the other. Along the path lies CYS101 as the most interactive residue, which we termed as the hub residue. The important residues along this path are shown in Figure 6 for 1OF2, of the B*2709 allele. Differences on this path for the B*2705 alleles are summarized in Figures 7A–D for 1OGT. The roles of the residues shown in Figures 6 and 7 relating to the structural and functional features of this path are discussed in this section.

(1) Substitution of ASP116 for HIS116 in the B*2705 allele results in stronger bonds both between the peptide and the protein and between the residues of the protein in the neighborhood of 116. In Figure 6 for B*2709, HIS116 exhibits no interactions with other residues along the path. For B*2705, on the other hand, ASP116 shown for 1OGT in Figure 7A makes three hydrogen bonds with ARG5 of the peptide and one hydrogen bond with ASN97.

In other members of B*2705 and B*2709, interactions other than the ones shown in Figures 6 and 7 are also present. In molecular dynamics simulations by Starikov et al. [21], for example, a salt bridge between LEU9 of the peptide and ASP116 in B*2705 was observed to limit the relative motions of these two residues. This causes changes along the interaction path and makes the system B*2705 becomes more fragile against nontrivial rearrangements, in parallel with recent findings on graphs [1]. Apart from differences in the important residues on the pathway predicted by various works the major common finding relates to the effects of replacing HIS116 in B*2709 with ASP116 on B*2705.

(2) Our calculations show that ARG6 exhibits strong response to external perturbation in B*2705's compared to those in B*2709. This implies that ARG6 is more rigidly embedded in its surroundings in B*2705. In Figure 6, ARG6 of 1OF2 is observed to make a single hydrogen bond directly with TYR99. In B*2705, however, ARG6 makes more bonds to its neighbors. In Figure 7B for 1OGT, for example, ARG6 and its neighbor MET6 are hydrogen bonded to CYS101, which in turn is covalently bonded to CYS164. Similarly, in Figure 7C, ARG6 and its neighbor TYR7 are hydrogen bonded to TYR27. In Figure 7D, ARG6 is observed to be an element of a cycle that is a loop of hydrogen bonded elements. This loop contains the residues in clockwise order: ARG6, MET5, CYS101, CYS164, GLU163, bridged by ARG1, ARG2, LYS3 of the peptide, followed by TYR99, TYR 7, terminating with ARG6. All of the residues along this loop are identified by the GNM and the maximum eigenvalue method. Perturbation of B*2705 and B*2709 at ARG6 results in a strong change in the correlations of CYS101 with its neighbors. The response of CYS101 is stronger in B*2705. Comparative energy calculations reported above, show that the interactions of ARG6 with its environment is stronger in B*2705 than in B*2709.

(3) Although ASN97 is not identified as a path member by the maximum eigenvalue method, this residue is shown in Figure 6 to make a hydrogen bond with TYR99. Although the latter does not show up as a path member, it makes a short loop of hydrogen bonding with ASN97 which is expected to reinforce the binding pocket. In Figure 7A, ASN97 is observed to make a hydrogen bond with HIS9 and with ARG5 of the peptide, thereby accentuating the tight binding of the peptide in the B*2705 allele. The pocket region that contains HIS9 is referred to as the B-pocket. According to comparative energy calculations reported above, ASN97 shows energetic differences for the two alleles, being more strongly bound to its environment in B*2705 than in B*2709.

(4) There is a major difference between the binding modes of the peptides to B*2705 and B*2709. In B*2709, ARG1 and ARG2 of the peptide bind strongly to the B pocket and the rest of the peptide remains relatively flexible contributing to the entropic advantage. The residues of the peptide in the C terminal are subject to nonpolar interactions. These interactions allow for only a few residue types, thus restricting the number of different peptides to only a few. B*2705 on the other hand, is capable of forming bonds with a multitude of residues because of the presence of ASP116. Hence, several different peptides may bind to B*2705. Therefore binding is not specific to a few peptides. Furthermore, the stronger bonding in the B*2705's presented in Table 3 results in an enhanced entropy penalty.

More specifically, for the B*2709 allele shown in Figure 6, ARG1 of the peptide is hydrogen bonded to GLU163, and ARG2 of the peptide is hydrogen bonded to GLU45 and GLU63. For the B*2705 allele, more extensive hydrogen bonding is observed between the peptide and the protein: In Figure 7A, ARG5 of the peptide binds to ASN97 and ASP116; in Figure 7B, ARG1 of the peptide binds to GLU163; in Figure 7C, ARG2 of the peptide binds to THR24; in Figure 7D, ARG2 of the peptide is bonded to TYR7 and LYS3 of the peptide is bonded to TYR99. Peptide flexibility is observed only for the HLA-B*2709 [37]. This suggests an entropic control of peptide recognition. The constraints on the strongly bound peptides in B*2705 constitutes an entropy disadvantage, or an entropy penalty. This hypothesis is supported by thermodynamic data [37]. Figures 7A–D show that the peptide is capable of forming several hydrogen bonds with various residues of the protein. Among these, the interaction with ASP116 and ARG5 of the strong peptide binding capability of the B*2705 binding groove that we observed raises the possibility that B*2705 allele may be capable of binding various different peptides. On the contrary, B*2709 exhibits a limited peptide binding capacity. There is indeed significant amount of experimental work aimed at understanding the differences in binding capacities of the two alleles. B*2709 shows a high specific preference for ligands with nonpolar C-terminal residues. The reason for this is the lack of ASP116. B*2705 accepts other residues at this position [38]–[39]. This is one reason of the ligand specificity of the B*2709's.

(5) In Figure 6, GLU163 is hydrogen bonded to ARG1 of the peptide. Its neighbor CYS164, is also shown in Figure 6. The corresponding conformation for the HLA-B*2705 allele is presented in Figure 7B. In both cases, the crystal structures show that CYS164 is covalently bonded to CYS101. Thus, in both alleles, the gate residue GLU163 can transfer the effects of the peptide to the rest of the protein through the bridge over the CYS164-CYS101 pair. We found that CYS101 is the residue that is strongly correlated with several other residues of the protein. In this sense, we call it the hub residue that controls the function of the protein. The present analysis shows that perturbation at the peptide binding site affects the behavior of CYS101. As shown in Figure 4, this response is stronger in B*2705 when compared with B*2709. A decrease of correlations of CYS101 is expected to result in an important change in the behavior of the protein. Warburton et al. mutated the residue CYS101 in another HLA Class I protein by replacing the CYS with SER, denoted by C101S mutation [40]. Due to the loss of the disulfide bond between CYS101 and CYS164 located between the alpha-helix and beta-sheet portions of the alpha2 domain of the A protein (heavy chain), the proteins lost stability and function.

(6) The stronger response of CYS101 to perturbations is a consequence of the strong inter-residue interactions around the binding region. The presence of ASP116 in B*2705 leads to strong inter-residue interaction. On the contrary, the presence of HIS116 in B*2709 makes the protein more flexible due to weaker interactions in the F pocket [21]. This socket is the region that contains the residues 114 and 116 and accommodates the carboxy terminus of the bound peptide.

(7) In Figure 6, the CO group of ASN97 is seen to make a hydrogen bond with the backbone NH of TYR99. The importance of this hydrogen bond for the stability of the protein has been shown by Blanco-Gelaz et al. [22] In that work, ASN97 was mutated to ASP97 which prevented the protein from gaining a stable conformation.

(8) As a general rule, if a residue is strongly coupled to its environment, then it leads to stronger response when its environment is perturbed, for example, when the residue is replaced with another amino acid of different size. It is therefore expected that when a protein exhibits strong inter-residue interaction energies at a given site, then it is less stable against external perturbations. This parallels the reasoning behind the stability of graphs [1], [8], [41].

(9) In Figure 7, we see that TYR27 makes a hydrogen bond with TYR63 of the beta-2-microglobulin. This is true for both the B*2705 and B*2709 alleles. Interaction of the heavy chain with the light chain is known to be a necessary determinant of stability, and any change in this interaction may be one reason for the misfolded or unfolded protein response [42]. However, although TYR27 appears as a significant residue on the interaction pathway, perturbation of the structure presented in Figures 4 and 5 does not induce a strong response in TYR27. The contribution of TYR27 to the unfolded protein response may not therefore be significant. However, although misfolding is associated with the activity of the peptide, the possible role of the B pocket of the heavy chain in unfolding has not been discarded [43]. The B pocket contains ARG6, TYR7, HIS9, THR24, GLU45, GLU63, and TYR99. The hub residue CYS101 makes two hydrogen bonds, one with MET5 and the other with ARG6, and ARG6 in turn makes two hydrogen bonds with TYR27.

There are two different lines of thought or hypothesis from the patogenetic perspective in the association of ankylosing spondylitis disease with HLA-B27 alleles [44]–[50]:

1. The arthritogenic peptide hypothesis assumes that the disease-causing specific (arthritogenic) peptides can bind on B*2705 but not on B*2709 and cause problems through recognition of HLA+peptide complexes expressed on the cell surface by the pathogenic CD8+ lymphocytes with their specific receptors. Therefore, this hypothesis suggests that the difference between B*2705 and B*2709 results from their peptide cargo loaded in the endoplasmic reticulum and their ability to stimulate immune cells on the cell surface. The ASP116 of B*2705 allows binding of a larger number of different peptides including the arthritogenic peptide, whereas only a limited number of peptides can bind to B*2709 which are not suitable to stimulate pathogenic autoreactive CD8+ lymphocytes.
2. The unfolded protein response hypothesis: Heavy chain of the HLA-B27 protein has a tendency to misfold within the endoplasmic reticulum because of its slow folding properties. Structural instability of B*2705 may increase its tendency to develop misfolded or unfolded forms of heavy chains, and increased accumulation of unfolded or misfolded heavy chains in the endoplasmic reticulum induces a specific type of inflammatory response known as unfolded protein response (UPR).

Our model shows a difference between the structural stability between B*2705 and B*2709 but also corroborates both hypothesis together by suggesting a role of binding peptides on the stability of structure. Due to strong interactions between the peptide and B*2705, specifically the presence of ARG at positions 2 and 5 in the ligand, this allele of HLA-B27 protein can bind a multitude of different peptides. Strong binding of these peptides influences the stability of the protein through interactions extending from residues ARG6 and TYR7 all the way to CYS101. The interaction between certain peptides and B*2705 heavy chain may result in the enhanced folding problems and an inflammatory reaction due to unfolded protein response. B*2709 on the other hand is highly selective for the peptides, having only a binding residue in the F-pocket, the B-pocket being rather floppy, leading to stable binding if the peptide is extremely suitable for this purpose. This selectivity may be an advantage for B*2709 allele by avoiding the binding of certain peptides which may increase the likelihood of structural instability.

## Supporting Information

Text S1 The model and formulation of the problem and the relative energy calculations.
(0.12 MB DOC)
