# Supplementary material for: Predicting Important Residues and Interaction Pathways in Proteins Using Gaussian Network Model: Binding and Stability of HLA Proteins
Source: PLoS Comput Biol. 2010 Jul 8;6(7):e1000845. doi: 10.1371/journal.pcbi.1000845 (PMC2900293; doi:10.1371/journal.pcbi.1000845)
Supplement: Text S1 — The model and formulation of the problem and the relative energy calculations. (0.12 MB DOC) [file pcbi.1000845.s001.doc]

**Text S1**

**The Model and Formulation of the Problem**

Here, we present the derivation of the main equations of the paper.

The protein exhibits energy, volume and residue position fluctuations. The probability distribution of the instantaneous values, $\hat{U}$, $\hat{V}$, and $\hat{\mathbf{R}}$, of the energy, volume and residue positions is given by the statistical - thermodynamic expression

(A1)

where, k is the Boltzmann constant and ***F*** is the force. The correlation of fluctuations $\left\langle\Delta\mathbf{R}_{\text{i}}\Delta\mathbf{R}_{\text{j}}^{T}\right\rangle$ of the i^th^ and j^th^ residues are defined as

(A2)

Using the expression, Eq. A1, for the distribution leads to [51]

$\left\langle\Delta\mathbf{R}_{i}\Delta\mathbf{R}_{j}^{T}\right\rangle=\text{kT} \left(\frac{\partial\mathbf{R}_{\text{i}}}{\partial\mathbf{F}_{\text{j}}}\right)_{\text{T,} \text{P,} \text{F i}\neq\text{j}}$ (A3)

In general, if Ф*_k_* represents any of the extensive variables *∆U,* ∆*V,* ∆***R***, and Ψ_k_ represent the conjugate variables *1/T,- P, F,* then, in principle, all higher moments of the extensive variables can be derived iteratively according to the rule [1]

$\left\langle\varphi\Delta\Phi_{k}\right\rangle=-k\frac{\partial}{\partial\Psi_{k}}\left\langle\varphi\right\rangle-k\left\langle\frac{\partial\varphi}{\partial\Psi_{k}}\right\rangle$ (A4)

where, $\varphi$ denotes the fluctuations of the extensive variables, ∆*U,* ∆*V,* ∆*R,* or their product of any order. For example, letting $\varphi=\Delta X_{i}$and $\Delta\Phi_{k}=\Delta X_{j}^{T}$, since $\left\langle\Delta X_{i}\right\rangle=0$, and

,

we obtain

$\left\langle\Delta X_{i}\Delta X_{j}^{T}\right\rangle=kT \left(\frac{\partial X_{\text{i}}}{\partial F_{\text{j}}}\right)_{\text{T,} \text{P,} \text{F i}\neq\text{j}}$ (A5)

Higher order moments can also be obtained by a recursion relation [1].

If we assume that the fluctuations result from a contact potential, then the force-position equation of state is $F=\Gamma R$ where, $\Gamma_{ij}$is the force constant matrix, whose ij’th element is taken as a constant *γ *if residue i and residue j of the protein are within a cutoff distance of $r_{c}$. If the distance is larger than $r_{c}$, the ij’th element is zero. The diagonal element $\Gamma_{ii}$is defined as the negative sum of the i’th row. Use of Eq. A3 then leads to

$\left\langle\Delta R_{i}\Delta R_{j}\right\rangle=kT\left( \Gamma^{-1}\right)_{ij}$ (A6)

We can similarly show the correlation of the energy fluctuations with fluctuations of residue positions. This is important because a strong correlation $\left\langle\Delta U\Delta\mathbf{R}_{i}\Delta\mathbf{R}_{j}^{T}\right\rangle$ between the total energy uptake of the protein and the residues i and j directly points to the residues i and j that are active in this energy transfer. For this, we write the energy fluctuations for the harmonic system as

$\Delta U=F_{k}\Delta R_{k}=F_{k}F_{j}\left(\Gamma^{-1}\right)_{jk}$ (A7)

Here, summation is understood over repeated indices. This expression is based on the assumption that the system is in a state of ease when the residues are at their mean positions.

Choosing $\varphi=\Delta U\Delta R_{i}$ and using the recursion relation, Eq. A4, we have

$\left\langle\Delta U\Delta R_{i}\Delta R_{j}\right\rangle=kT\frac{\partial}{\partial F_{j}}\left\langle\Delta U\Delta R_{i}\right\rangle+kT\left\langle\frac{\partial}{\partial F_{j}}\left(\Delta U\Delta R_{i}\right)\right\rangle$ (A8)

We write the first term in angular brackets on the right hand side as

$\left\langle\Delta U\Delta R_{i}\right\rangle=kT\frac{\partial}{\partial F_{i}}\left\langle\Delta U\right\rangle+kT\left\langle\frac{\partial\Delta U}{\partial F_{i}}\right\rangle=0+kT\left\langle\frac{\partial\left(F_{k}\Delta R_{k}\right)}{\partial F_{i}}\right\rangle$ (A9)

$$=kT\left\langle\delta_{ik}\Delta R_{k}+F_{k}\frac{\partial\Delta R_{k}}{\partial F_{i}}\right\rangle=0+kT\left\langle F_{k}\frac{\partial\Delta R_{k}}{\partial F_{i}}\right\rangle=kT\left\langle F_{k}\Gamma_{ik}^{-1}\right\rangle$$

$$=kT\left\langle\Delta R_{i}\right\rangle=0$$

The second term is written as

$$kT\left\langle\frac{\partial}{\partial F_{j}}\left(\Delta U\Delta R_{i}\right)\right\rangle=kT\left\langle\Delta U\frac{\partial\Delta R_{i}}{\partial F_{j}}\right\rangle+kT\left\langle\Delta R_{i}\frac{\partial\Delta U}{\partial F_{j}}\right\rangle$$

$=kT\mathbf{\Gamma}_{ij}^{-1}\left\langle\Delta U\right\rangle+kT\left\langle\Delta R_{i}\Delta R_{j}\right\rangle$ (A10)

Since $\left\langle\Delta U\right\rangle=0$,

$\left\langle\Delta U\Delta\mathbf{R}_{i}\Delta\mathbf{R}_{j}^{T}\right\rangle=kT\left\langle\Delta\mathbf{R}_{i}\Delta\mathbf{R}_{j}^{T}\right\rangle$ (A11)

**The relative energy calculations**

The approximate estimates of interaction energies are performed by static minimization of the energies of the system. The energy minimizations are performed for the native protein, in the absence of explicit water, using OPLS force field (2, 3) and Hyperchem. The dielectric constant is taken with a scale factor of unity, the 1-4 electrostatic scale factor is taken as 0.5 and the van der Waals parameter is taken as 0.25, with cutoff switched off. The OPLS energy function contains bond, angle, torsion, non-bonded, electrostatic and hydrogen bond components. The minimization is performed by the Polack-Ribiere conjugate gradient algorithm, with a termination condition of RMS gradient of 0.1 kcal/(Å mol).

In the calculations, we first minimized the energy of the system. In order to calculate the interaction of a given residue with the rest of the protein, which is in the minimum energy conformation, the residue is chosen in the matrix of the remaining residues that are kept in their native states, and the interaction energy of the chosen residue is minimized around the given conformation using the OPLS force field. In doing this calculation only the residue of interest is left flexible and the conformations of the remaining protein residues are kept fixed at their native values. The OPLS force field is optimized for biological systems, yet the other force fields, MM+, BioCharmm, and Amber give similar results. The calculated energies are sensitively dependent on the value of the dielectric constant chosen. In calculations of biological systems in the absence of explicit water, its value is taken between 1 and 4.

The interaction energy of the peptide with the protein (the heavy chain A) is calculated in three steps: (i) the minimum energy$E\left(i\right)$of the peptide and the protein together is calculated; (ii) the energy$E\left(ii\right)$of the protein only, kept rigid in the conformation found in step (i), the co-crystal chain A structure, is calculated; (iii) the energy $E\left(iii\right)$of the peptide only, kept rigid in the conformation found in step (i), the co-crystal peptide structure, is calculated. The interaction energy of the peptide is taken as$E\left(i\right)-E\left(ii\right)-E\left(iii\right)$. This calculation gives an idea about energy changes in rigid docking, and should not be taken as the binding energy in the thermodynamic sense. The energy of GLU163 given in Table 3 is calculated on the same basis.

On the other hand, mutations are performed by using the ‘mutate’ command of Hyperchem which replaced the wild type residue by the residue of interest. The interaction energy of the residue of interest with its surroundings in the energy minimized unmutated and mutated protein is determined.

**References**

1. Callen HB (1985) Thermodynamics and an introduction to thermostatistics: Wiley.

2. Jorgensen WL, Maxwell DS, TiradoRives J (1996) Development and testing of the OPLS all-atom force field on conformational energetics and properties of organic liquids. J Am Chem Soc 118: 11225-11236.

3. Rizzo RC, Jorgensen WL (1999) OPLS all-atom model for amines: Resolution of the amine hydration problem. J Am Chem Soc 121: 4827-4836.
